# Patterns of genetic variation and the role of selection in *HTR1A* and *HTR1B* in macaques (*Macaca*)

**DOI:** 10.1186/s12863-014-0116-5

**Published:** 2014-11-07

**Authors:** Milena R Shattuck, Jessica Satkoski-Trask, Amos Deinard, Raul Y Tito, David G Smith, Don J Melnick, Ripan S Malhi

**Affiliations:** Department of Anthropology, University of Illinois, Urbana, IL USA; Department of Anthropology, University of California, Davis, CA USA; Department of Anthropology, University of Minnesota, Minneapolis, MN USA; California National Primate Research Center, Davis, CA USA; Department of Ecology, Evolution, and Environmental Biology, Columbia University, New York, NY USA; Department of Anthropology, Animal Biology, and Institute for Genomic Biology, University of Illinois, Urbana, IL USA

**Keywords:** Serotonin, *Macaca*, Molecular evolution, *HTR1A*, *HTR1B*, Genetic variation

## Abstract

**Background:**

Research has increasingly highlighted the role of serotonin in behavior. However, few researchers have examined serotonin in an evolutionary context, although such research could provide insight into the evolution of important behaviors. The genus *Macaca* represents a useful model to address this, as this genus shows a wide range of behavioral variation. In addition, many genetic features of the macaque serotonin system are similar to those of humans, and as common models in biomedical research, knowledge of the genetic variation and evolution of serotonin functioning in macaques are particularly relevant for studies of human evolution. Here, we examine the role of selection in the macaque serotonin system by comparing patterns of genetic variation for two genes that code for two types of serotonin receptors – *HTR1A* and *HTR1B* – across five species of macaques.

**Results:**

The pattern of variation is significantly different for *HTR1A* compared to *HTR1B*. Specifically, there is an increase in between-species variation compared to within-species variation for *HTR1A*. Phylogenetic analyses indicate that portions of *HTR1A* show an elevated level of nonsynonymous substitutions. Together these analyses are indicative of positive selection acting on *HTR1A*, but not *HTR1B*. Furthermore, the haplotype network for *HTR1A* is inconsistent with the species tree, potentially due to both deep coalescence and selection.

**Conclusions:**

The results of this study indicate distinct evolutionary histories for *HTR1A* and *HTR1B*, with *HTR1A* showing evidence of selection and a high level of divergence among species, a factor which may have an impact on biomedical research that uses these species as models. The wide genetic variation of *HTR1A* may also explain some of the species differences in behavior, although further studies on the phenotypic effect of the sequenced polymorphisms are needed to confirm this.

**Electronic supplementary material:**

The online version of this article (doi:10.1186/s12863-014-0116-5) contains supplementary material, which is available to authorized users.

## Background

Over the past 50 years, research has increasingly highlighted the role of the serotonin system in shaping behavior (for reviews see [1-4]). Genetic, pharmaceutical, and hormonal studies have shown that, across taxa, serotonin influences age at dispersal [5,6], social behavior [7-10], exploratory behavior [11], and aggressive behavior [12-16], among others. Within humans, it has been shown to play a role in a number of psychological conditions including alcoholism [17,18], anxiety [19], depression [20,21], and schizophrenia [22], and many psychiatric medications work by targeting components of the serotonin system. Thus, a detailed understanding of the serotonin system, including its genetic variation and evolution, has wide reaching implications for both biomedical research and for understanding behavioral evolution (see, for example, [16]). However, despite the large amount of research devoted to this system, it is rarely placed in an evolutionary context.

Macaques (*Macaca*) represent a useful model for addressing the evolution of the serotonin system. Macaques split from other cercopithecine primates approximately 6–8 million years ago (mya); shortly thereafter they migrated from Africa to Asia (approximately 5.5 mya) where they experienced an adaptive radiation [23-25]. There are 19–22 extant species known today [26,27], and a few extinct species are known from the fossil record [23]. While closely related, these species exhibit diverse social behaviors, with some species showing relaxed hierarchies, low levels of severe aggression, and high rates of reconciliation, and others showing strict hierarchies, high levels of severe aggression, and low rates of reconciliation; other behaviors that vary include age at dispersal, mothering styles, degree of kin affiliation, and social play patterns [28,29], all of which are likely influenced by serotonin. These differences among species are significantly influenced by phylogeny [28,29], suggesting that genetic differences in neurological functioning may underlie the behavioral differences in this genus [30]. We therefore know that there have been multiple evolutionary events that have driven the diversification of this genus and we might expect to see evolutionary signatures of these events in the genetics of serotonin and other neurological systems.

Moreover, macaques also share interesting parallels with humans. Specifically, they occupy a wide range of habitats, from snowy temperate zones to tropical forests to urban settings, making macaques – particularly the rhesus macaque (*Macaca mulatta*) – the most widely distributed of nonhuman primates. It has been hypothesized that the exploitation of a large part of the world by both humans and macaques was the result of the evolution of similar behavioral strategies, such as high between-group aggression [31,32]. Such behaviors are often related to serotonin functioning [5,6,12-15] and are hypothesized to comprise a "behavioral syndrome" characteristic of invasive species [33-35]. In line with this theory, several similar, though independently evolved, genetic variants related to serotonin functioning have been identified in both rhesus macaques and humans, most notably *SLC6A4* [36] and *MAOA* [13], and this has been argued to be the result of similar selective pressures acting on the serotonin system [31]. The repeated recruitment of the same neurological system to produce similar behaviors, what we call here parallel evolution *sensu* Haldane [37], may explain behavioral similarities across a wide range of animals [38,39]. Thus, understanding the evolution of the serotonin system in macaques may grant insight into the evolution of this system in humans.

Most studies of the serotonin system have focused on the serotonin transporter gene (*SLC6A4*), but unfortunately this has led to the neglect of other potentially important genes. Two such genes are *HTR1A* and *HTR1B*. These two genes code for serotonin receptor types 1A and 1B, and are located on chromosomes 4 and 6 of the macaque genome, respectively [40]. They are similar in structure, having just one exon (1,269 and 1,173 base pairs (bp) for *HTR1A* and *HTR1B*, respectively) and no introns; both receptor types are associated with behavior [11,41-45]. While similar in structure and exhibiting an overlap in function [46], experiments with knockout mice indicate that these two receptors modulate behavior in opposite ways [11,41], potentially due to the different distributions of the receptors within the brain [47]. Knockout mice lacking *HTR1A* show increased anxiety and decreased exploratory behavior compared to the wildtype [11]; in contrast, knockout mice lacking *HTR1B* are more impulsive, less anxious, and more aggressive than the wildtype [41]. Thus, differences occurring in the coding and regulatory regions of these receptor genes may contribute to the diverse behaviors macaque species exhibit today.

Here, we begin to place *HTR1A* and *HTR1B* in an evolutionary context by investigating the pattern of genetic variation of these genes within and among several species of macaques and testing for the role of selection in these species. By using this approach, we hope to identify the targets of selection, including potentially important mutations that separate species and may contribute to the diversity of macaque behavior. However, as a strictly genetic study, this is merely a first step in examining the evolution of serotonin in macaques; further studies would include directly examining the phenotypic effects of these mutations.

## Results

### Molecular diversity

Overall, indices of genetic diversity are comparable to those reported for other areas of the macaque genome (Tables 1 and 2; see also Additional file 1: Table S5) [48]. Several studies have found a major split in the *M. mulatta* lineage between Chinese and Indian populations [24,49-53]. Because the presence of population substructure might have an effect on analyses, we examined representatives of each branch of this species separately. The values for all genetic diversity indices (Tables 1 and 2) are very similar for both lineages, and for all analyses separate examination of the two *M. mulatta* branches did not affect results. Therefore, for the remainder of the paper, we only report the results for the species as a whole.

**Table 1:** **Indices of within-species genetic diversity found in** ***HTR1A*** **for five species of macaque**

	**Mul** ^**a,b**^			**Fas** ^**a,c**^		**Fus** ^**a**^	**Nem** ^**a**^	**Syl** ^**a**^
	China	India	Total	Orig.	Mod.			
Polymorphisms	17	15	18	28	7	6	27	2
SNP	15	13	16	26	7	6	25	2
Indel	2	2	2	2	0	0	2	0
Coding (1269 bp)	3	3	4	4	0	2	5	1
Nonsynonymous	0	0	0	2	0	1	1	0
Noncoding (1850 bp)	14	12	14	22	7	4	22	1
Theta (S)	4.65	3.57	3.76	7.13	1.97	1.65	8.28	0.77
Theta (π)	5.72	3.28	4.65	5.82	2.27	1.82	9.95	1.07
Tajima's D	−0.78	0.28	0.25	−0.88	0.49	0.33	0.46	1.03
p-value	> 0.1	> 0.1	> 0.1	> 0.1	> 0.1	> 0.1	> 0.1	> 0.1

^a^Mul: *M. mulatta*; Fas: *M. fascicularis*; Fus: *M. fuscata*; Nem: *M. nemestrina*; Syl: *M. sylvanus*.

^b^For *M. mulatta*, indices for both the Chinese and the Indian populations are shown separately, as well as indices for the species as a whole.

^c^For *M. fascicularis*, indices for *HTR1A* are shown both with and without the outlier.

**Table 2:** **Indices of within-species genetic diversity found in** ***HTR1B*** **for five species of macaque**

	**Mul** ^**a**^			**Fas** ^**a**^	**Fus** ^**a**^	**Nem** ^**a**^	**Syl** ^**a**^
	China	India	Total				
Polymorphisms	8	10	11	12	4	5	2
SNP	7	10	10	11	4	5	2
Indel	1	0	1	1	0	0	0
Coding (1173 bp)	3	3	3	2	4	4	2
Nonsynonymous	0	0	0	0	2	1	0
Noncoding (944 bp)	5	7	8	10	0	1	0
Theta (S)	2.04	2.74	2.35	3.02	1.10	1.66	0.77
Theta (π)	2.25	3.77	3.20	3.51	1.74	1.35	0.68
Tajima's D	−0.24	1.27	0.84	0.28	1.65	−0.68	−0.45
p-value	> 0.1	> 0.1	> 0.1	> 0.1	> 0.1	> 0.1	> 0.1

^a^See Table 1 for explanation.

**Figure 1:**
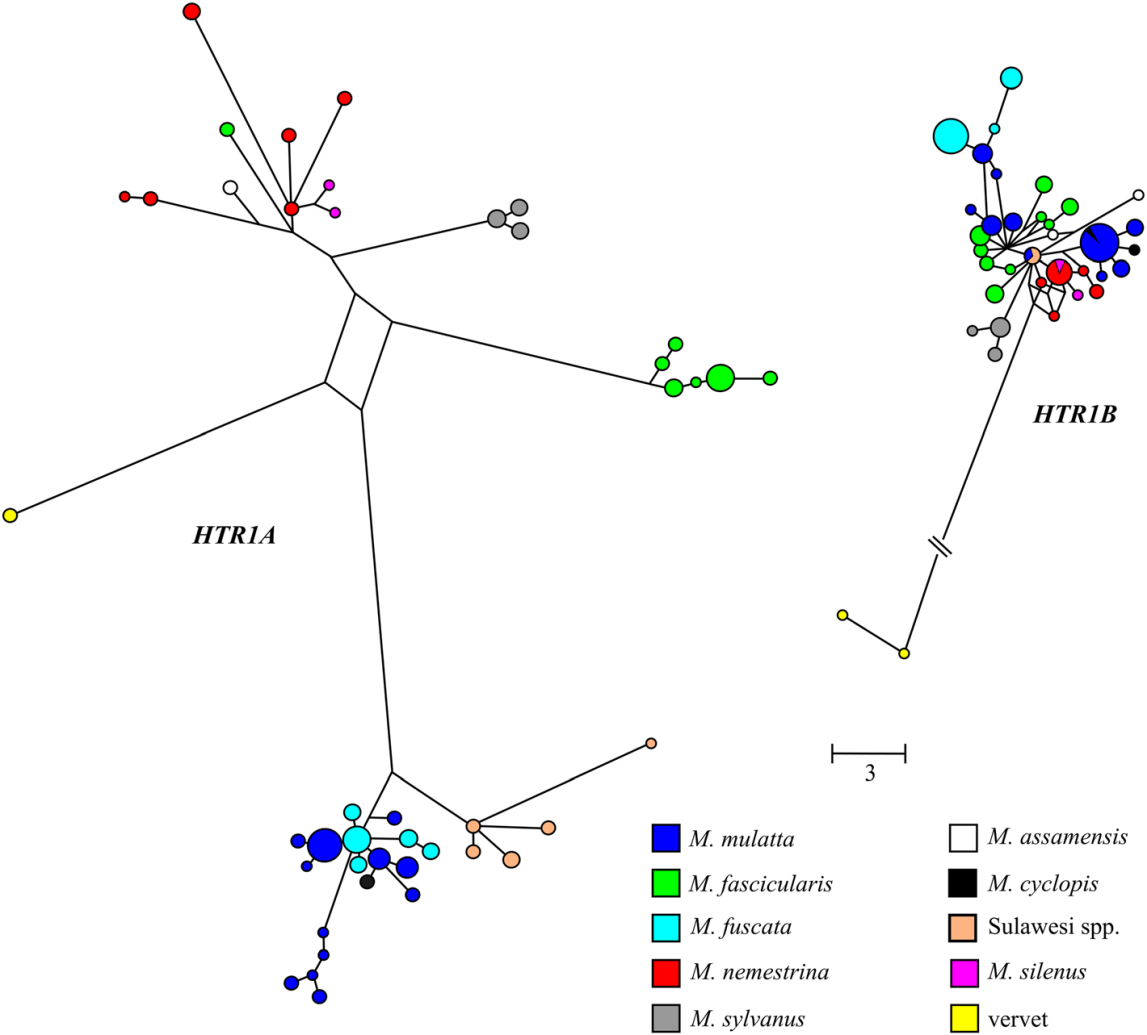
**Shortest unrooted haplotype networks for** ***HTR1A*** **(left) and** ***HTR1B*** **(right).** The networks represent all areas sequenced: coding regions and the areas flanking either side of the gene, including potential regulatory regions. Each circle represents a haplotype whose size is proportional to the frequency of the haplotype. The lengths of the lines connecting the circles are proportional (according to the scale provided) to the number of mutations that separate each haplotype. Because of the larger number of mutations separating the vervet from the macaques in *HTR1B*, this line (which represent 52 mutations) is not drawn to scale. Sulawesi species include *M. nigra*, *M. tonkeana*, and *M. maura*. For *HTR1B*, only the original *M. nigra* is shown (see Methods and Results).

**Figure 2:**
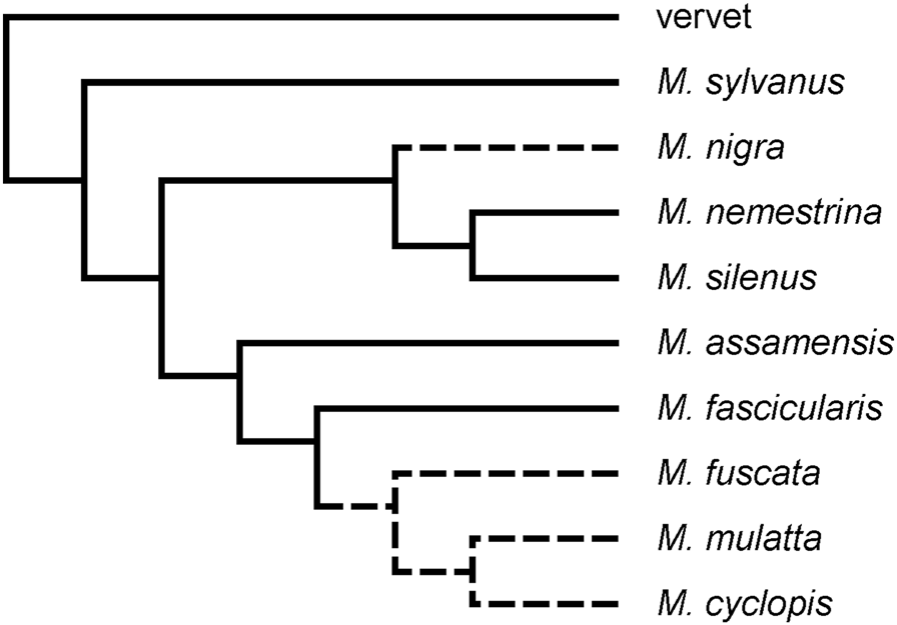
**Phylogeny used to run analyses in PAML, which is based on a number of previous molecular studies** [62]**.** The dashed lines indicate the species in which we found both the loss of a codon and two identical nonsynonymous mutations. Based on this phylogeny, these mutations are found in two distinct lineages – the *mulatta* group and the Sulawesi group (of which *M. nigra* is a representative) – and is likely a result of deep coalescence.

A comparison of the two genes shows that genetic distances between species for *HTR1A* are generally high compared to *HTR1B* (Table 3). The only exception to this is between *M. mulatta* and *M. fuscata*, where the divergence is actually lower in *HTR1A* than *HTR1B*. These trends are also reflected in the gene trees (Figure 1, which includes both coding and noncoding regions; for coding region only, see the Additional file 1: Figure S1) by both the wider spacing of the haplotypes in *HTR1A* than in *HTR1B* and the tight clustering of *M. mulatta*, *M. fuscata*, and *M. cyclopis* in *HTR1A*. Because these three species are members of a monophyletic group that excludes the other macaque species studied (Figure 2), for ease of future discussion, *M. mulatta*, *M. fuscata*, and *M. cyclopis* will be referred to as the *mulatta* group (see [24,49,50]).

**Table 3:** **Genetic distance within and among species for** ***HTR1A*** **and** ***HTR1B***

***HTR1A*** ^**a,b,c**^					
	Mul	Fas	Fus	Nem	Syl
Mul	0.0015				
Fas	0.0125	0.0019			
Fus	0.0013	0.0120	0.0006		
Nem	0.0122	0.0068	0.0121	0.0032	
Syl	0.0115	0.0083	0.0115	0.0060	0.0003
***HTR1A*** **(outlier removed)** ^**a,b,c**^					
	Mul	Fas	Fus	Nem	Syl
Mul	0.0015				
Fas	0.0125	0.0007			
Fus	0.0013	0.0121	0.0006		
Nem	0.0122	0.0071	0.0121	0.0032	
Syl	0.0115	0.0085	0.0115	0.0060	0.0003
***HTR1B*** ^**a,b,c**^					
	Mul	Fas	Fus	Nem	Syl
Mul	0.0015				
Fas	0.0021	0.0017			
Fus	0.0032	0.0036	0.0008		
Nem	0.0018	0.0020	0.0038	0.0006	
Syl	0.0028	0.0028	0.0047	0.0023	0.0003

^a^Diagonal elements show the nucleotide diversity within species and the off-diagonal elements show the nucleotide diversity among species. Nucleotide diversity averaged over all loci.

^b^See Table 1 for species names.

^c^See Table S4 (Additional file 1) for similar comparisons in the nonfunctional regions sequenced.

While members of the same species tend to cluster together in the *HTR1A* haplotype network, one *M. fascicularis* individual provides an exception to this pattern by showing greater similarity to a clade with *M. nemestrina*, *M. assamensis*, and *M. silenus* individuals than to the other *M. fascicularis* individuals (Figure 1). It shares none of the SNPs that distinguish the other *M. fascicularis* individuals and possesses several SNPs that are unique to this individual. We repeated sequencing on this individual in order to rule out a PCR or sequencing error. This individual likely represents either substructure within *M. fascicularis* or introgression. It was not clear how this individual would affect our results, so we report here the results of analyses of *HTR1A* with and without this outlier (Tables 1, 3, and 4). With the exception of the HKA test, inclusion or exclusion of the outlier did not significantly change the test results. Because this individual was largely undifferentiated in *HTR1B*, its inclusion or exclusion did not significantly alter the results. Therefore, for *HTR1B*, we only report the results of the original dataset, which includes the outlier.

**Table 4:** **Results of two selection tests for** ***HTR1A*** **and** ***HTR1B***

***HTR1A*** ^**a,d**^											
**ω** ^**b**^						HKA^**c**^					
	Mul	Fas	Fus	Nem	Syl		Mul	Fas	Fus	Nem	Syl
Mul	—	**0.032**	**0.040**	**0.015**	**0.013**	Mul	—	0.107	0.939	**0.008**	0.108
Fas	**0.242**	—	**0.038**	0.195	0.115	Fas	13.93	—	0.269	0.210	0.676
Fus	**0.046**	**0.252**	—	**0.020**	**0.017**	Fus	3.39	10.72	—	**0.033**	0.512
Nem	**0.152**	0.423	**0.162**	—	**0.035**	Nem	**22.15**	10.23	**18.89**	—	0.449
Syl	**0.121**	0.269	**0.129**	**0.043**	—	Syl	14.82	5.84	6.93	6.22	—
***HTR1A*** **(outlier removed)** ^**a,d**^											
**ω** ^**b**^						HKA^**c**^					
	Mul	Fas	Fus	Nem	Syl		Mul	Fas	Fus	Nem	Syl
Mul	—	**0.033**	**0.038**	**0.013**	**0.013**	Mul	—	**0.004**	0.939	**0.008**	0.108
Fas	**0.253**	—	**0.037**	0.229	0.150	Fas	**23.04**	—	**0.008**	**0.014**	0.115
Fus	**0.046**	**0.263**	—	**0.014**	**0.014**	Fus	3.39	**22.69**	—	**0.033**	0.512
Nem	**0.152**	0.460	**0.162**	—	**0.045**	Nem	**22.15**	**19.06**	**18.89**	—	0.449
Syl	**0.121**	0.296	**0.129**	**0.043**	—	Syl	14.82	13.84	6.93	6.22	—
***HTR1B*** ^**a,d**^											
**ω** ^**b**^						HKA^**c**^					
	Mul	Fas	Fus	Nem	Syl		Mul	Fas	Fus	Nem	Syl
Mul	—	**0.028**	**0.032**	**0.035**	**0.017**	Mul	—	0.968	0.804	0.763	0.072
Fas	**0.000**	—	0.082	**0.027**	0.061	Fas	1.75	—	0.992	0.201	0.250
Fus	**0.128**	0.180	—	0.065	**0.043**	Fus	5.25	2.21	—	0.286	0.346
Nem	**0.039**	**0.076**	0.189	—	**0.047**	Nem	4.73	7.58	9.86	—	0.245
Syl	**0.000**	0.000	**0.113**	**0.031**	—	Syl	14.53	10.41	8.78	9.34	—

^a^See Table 1 for list of species.

^b^Numbers in the lower left diagonal are the ratio of nonsynonymous to synonymous substitutions calculated between species and numbers in the upper right are their respective p-values.

^c^Lower left diagonal shows the sum of deviations calculated in the HKA program and the upper right shows their respective p-values.

^d^Numbers bolded have a p <0.05.

### Evolutionary analyses

For our first set of tests, we applied the HKA test using pairwise comparisons for all species for which we had multiple samples (see Methods). This test compared the levels of within-species variation to between species variation across several loci (see Methods and the Additional file 1: Table S3 for a list of the loci used). We obtained significant results for some between-species comparisons of *HTR1A*, but not for any comparisons of *HTR1B* (Table 4). These results were mainly driven by a high level of between-species divergence in *HTR1A* when compared to the additional loci sequenced (Figure 1; see also Additional file 1: Table S4), and are suggestive of positive selection occurring in macaques. HKA results were not significant for any comparison with *M. sylvanus*, nor for the comparison between *M. mulatta* and *M. fuscata*. All other between-species comparisons are significant for *HTR1A* when the outlying *M. fascicularis* individual is excluded. When the outlier is included, only comparisons between *M. nemestrina* and the *mulatta* group (*M. mulatta* and *M. fuscata*) remain significant. Repeating these analyses using only the coding region of the gene largely replicated these results (Additional file 1: Table S6), although comparison between the *mulatta* group and *M. sylvanus* became significant. Likewise, for *HTR1B,* for some comparisons with *M. sylvanus* the HKA results for the coding region alone were significant, although in this case this was due to higher than expected diversity *within M. sylvanus*. For comparisons with *Chlorocebus aethiops*, see the Additional file 1: Table S7 and Further Discussion.

Our second set of tests focused on the ratio of synonymous to nonsynonymous substitutions (**ω**). The overall ratios for both genes (Table 4) were almost all either significantly lower than one or else approaching significance. This is consistent with previous studies that have shown the serotonin system to be evolutionarily conserved [54,55]. However, an examination of **ω** alone is known to be a highly conservative test, particularly if only a small portion of the entire gene is under selection (see Methods). Therefore, we used phylogenetic analyses using maximum likelihood (PAML), which assigns likelihood values to different models of evolution. Models that incorporate positive selection can be compared to null models and a likelihood ratio test (LRT) used to determine which model best fits the data. Two types of models were compared: variable sites and variable branch. In the variable sites models, **ω** was allowed to vary among sites; the null models (M1a, M7, and M8a) did not allow for any site to have **ω > 1**, whereas the selection models (M2a and M8) did. The LRT showed significant results for *HTR1A* (p <0.001 for all tests), but not for *HTR1B* (p >0.1 for all tests; Table 5). Bayes empirical Bayes analyses for both M2a and M8 classified codon 15 in *HTR1A* as a site where **ω** >1.0 with a high posterior probability (99.2%), indicating that this site of *HTR1A* was possibly under selection even though the overall **ω** was low. Two other sites approached significance: codon 22 (with posterior probability of 93.8 and 94.5% for M2a and M8, respectively) and codon 329 (posterior probability of 93.6 and 94.4%). The variable branch models tested to see whether **ω** varied among branches. The LRT using the variable branch models were non-significant for both genes (p >0.1 for all tests; not shown); that is, no selection was detected on one specific lineage.

**Table 5:** **Results of the PAML analyses comparing various sites models for** ***HTR1A*** **and** ***HTR1B***

**Models compared**		**p-values**	
Null^a,c^	Selection^b,c^	*HTR1A*	*HTR1B*
M1a	M2a	0.0007	0.9909
M7	M8	0.0007	0.9160
M8a	M8	0.0001	0.8911

^a^M1, M7, and M8a represent null models (no selection).

^b^M2a and M8 allow for selection at some sites within the gene.

^c^See [95,96] for detailed explanation of models.

Finally, we calculated the Tajima’s D value for both *HTR1A* and *HTR1B*, which are shown in Tables 1 and 2. The Tajima’s D values were non-significant (p >0.1) for both genes for all species analyzed.

### The placement of *M. nigra*

Although not closely related to the *mulatta* group, the haplotype network of *HTR1A* shows that *M. nigra* clusters with these species, sharing several derived mutations unique to the *mulatta* group (Figures 1 and 2). Notably, all four species – *M. mulatta*, *M. fuscata*, *M. cyclopis*, and *M. nigra* – share both a codon loss (codon 33) and two nonsynonymous substitutions (codons 15 and 22) that no other macaque species exhibit. Based on models of the human serotonin receptor [56], all three mutations affect the extracellular amino terminal region of the receptor. The effect of the codon loss is unknown, however, one nonsynonymous substitution is identical to a polymorphism identified in humans, Gly22Ser [57]. While this polymorphism has not been associated with behavior, possibly due to its low frequency in humans [57,58], it does show a different pharmacological response than the wildtype [59].

Because it is unexpected for both the *mulatta* group and *M. nigra* to share these unusual mutations, especially the loss of a codon, to the exclusion of other, more closely related species (e.g., *M. nemestrina*), for *HTR1A* we verified these results by sequencing two additional *M. nigra* individuals, as well as one individual each from *M. maura* and *M. tonkeana*, using the same methods outlined below. *M. nigra*, *M. maura*, and *M. tonkeana* are all located on Sulawesi and are closely related to each other [24], and so we collectively refer to these as the Sulawesi species. All four additional samples had the same codon loss and nonsynonymous mutations, and all cluster with the *mulatta* group (Figure 1).

### Recombination

We estimated the recombination rate as *C* = 0.002 for *HTR1A* and *C* <0.001 for *HTR1B* using the methods of Hey and Wakeley [60]. For *HTR1A*, these estimates seem to be largely the result of recombination outside the coding region for *HTR1A* because when we only included the coding region, we estimated *C* = 0, suggesting no recombination occurring within the coding region for *HTR1A* (Additional file 1: Figure S1).

## Discussion

Although behaviors are complex and no explanation of behavior can be reduced to a single gene or gene product, it is increasingly recognized that genetic variation plays an important role in mediating social behavior [9,38,39]. Because evolution is ultimately a genetic process, understanding behavioral evolution entails understanding the evolution of neurological systems and the genes that underlie them. The serotonin system represents one such genetic/neurological system. Its connection to behavior has been well established and may play a role in the evolution of the wide behavioral diversity found in macaques. Here, a window on the evolution of the serotonin system in macaques is provided by examining the evolutionary history of two serotonin related genes: *HTR1A* and *HTR1B*.

### A comparison of *HTR1A* and *HTR1B* and the possible role of selection

Compared to *HTR1B*, *HTR1A* interspecific variation is much higher. While all macaque species cluster tightly together in *HTR1B*, for *HTR1A*, three main groups of haplotypes can be discerned: one including *M. sylvanus*, *M. nemestrina*, *M. silenus*, and *M. assamensis* (with the outlying *M. fascicularis* individual), a second one consisting of *M. fascicularis*, and a third one that includes the *mulatta* group and the Sulawesi macaques. The difference between the two genes can also be seen in the nucleotide diversity among species, which is 2.5 to 6.5 times higher in *HTR1A* than in *HTR1B* (and in the additional nonfunctional regions sequenced; see the Additional file 1: Table S4). Most of this variation, both within and among species, is found outside of the coding region. An examination of the coding regions alone shows that, although *HTR1A* still shows more interspecific divergence, its difference with *HTR1B* is less pronounced.

Regardless, *HTR1A* and *HTR1B* appear to have experienced distinct evolutionary histories and our analyses suggest that different levels of selection on these two genes played a role. Overall **ω** for both genes was low, suggesting that purifying selection is the dominant force acting on both genes. However, phylogenetic analyses that allowed **ω** to vary among sites within the coding region of the gene provided evidence that positive selection is acting on *HTR1A*, but not *HTR1B*. That is, there is evidence that selection is acting to alter the structure of the protein itself, which is consistent with the finding of both a codon loss and two nonsynonymous substitutions in a subset of the macaque species.

The HKA tests also suggest that for *HTR1A*, but not *HTR1B*, non-neutral evolution is occurring. When the outlying *M. fascicularis* individual was removed, several between-species comparisons were significant for HKA due to the high level of between-species divergence in *HTR1A* compared to within-species variation, consistent with a positive selective sweep [61]. However, the HKA results were not significant for any comparison with *M. sylvanus*, or between *M. mulatta* and *M. fuscata. M. mulatta* and *M. fuscata* exhibit a low degree of differentiation from each other (Figure 1), due to their close phylogenetic relationship, which explains this non-significant result. *M. sylvanus* is the outgroup to all Asian macaque species, being an African relic species and the first to diverge from the other macaque species in any phylogenetic analysis [24,62,63]. The non-significant results for comparison to *M. sylvanus* may indicate that the evolutionary events driving the high level of between-species divergence occurred after the macaque radiation diverged from its African congeners and began to spread across Eurasia. Alternatively, the relatively low sample size for this species (2N = 8) could have contributed to this result. If the outlier is included, comparisons with *M. fascicularis* become non-significant because the estimate of within-species variation for this species increases dramatically. Even with the inclusion of the outlier, however, results remain significant between *M. nemestrina* and the *mulatta* group. These results were largely duplicated when considering only the coding region, even with the reduced variation (although see the Additional file 1: Further Discussion). This, in addition to the PAML results, suggests that natural selection has acted on structure of the *HTR1A*. However, because a large amount of noncoding variation contributes to the high interspecific diversity found in *HTR1A*, it is also possible that the function of regulatory regions has been altered as well, although this needs to be confirmed with additional studies.

Macaque species are known to differ greatly in behaviors such as aggression, dispersal, mothering style, and exploratory behavior, all of which have been shown to be modified by serotonin. Thus, we might expect to see a similar level of divergence in genes connected to serotonin. *HTR1A* shows such a divergence and may be affecting these behaviors in macaques. That this divergence seems to be the result of selection provides evidence that interspecific behavioral variation is not entirely constricted by phylogeny or driven by drift. In a previous study, Claw and colleagues [64] provided evidence that positive selection had occurred on the human lineage in two serotonin-related genes, *SLC6A4* and *HTR2C*, as indicated by an elevated rate of divergence from chimpanzees for the regulatory regions of these genes. While these are not the same genes examined here, they are a part of the general serotonin system, likely the broader target of selection, and it is therefore interesting to compare their results to ours. It has been suggested that parallel evolution of behavioral patterns, via the serotonin system, is occurring between humans and macaques [31] and potentially allowed both taxa to become widespread and adapt to a range of habitats [31,32]. While we do not address this directly, our results are consistent with this hypothesis and the evidence of positive selection in both taxa is intriguing and warrants further study.

### Non-selective evolutionary forces

Although all of the tests used here were designed to detect departures from non-neutral evolution, there are non-selective forces, such as demography and recombination, that may influence these tests; however, these factors seem unlikely to explain the overall results. For example, while demographic events may affect the HKA test [61], this test has also been argued to be somewhat robust to demographic assumptions [65] as demography is expected to have a similar effect on all loci and the HKA test employs a multilocus approach. In addition, Tajima’s D, which is known to be highly sensitive to demography [66], was not significant. That is, it is unclear why a past demographic event might be expected to affect only *HTR1A*, to the exclusion of *HTR1B* and the five nonfunctional loci, and why it would significantly affect the HKA test and not Tajima’s D. Furthermore, a demographic event is unlikely to produce the significant results found in PAML, as tests based on **ω** do not depend on demographic assumptions [61].

Another potential limitation of these analyses is that they are based on captive studies. In particular, the HKA is based on measures of within-species variation, and our estimate might be underestimated by using captive animals. This might be thought of as a specific example of a demographic effect as these populations have (artificially) undergone a bottleneck. We addressed this possibility by selecting nonrelatives from multiple geographic origins (see Methods) in an attempt to capture the true population parameter. However, even if the study design failed to capture the true extent of intraspecific genetic variation, as with other demographic effects, the HKA test should be robust to this problem as the use of captive animals should reduce estimation of within-species variation across all loci.

We did find evidence for recombination, which can affect analyses. This is a conservative assumption for the HKA test, such that the presence of recombination makes it *less* likely for it to detect selection. However, its presence can lead to an increase in false positives for the PAML comparing various sites models [67]. However, two factors make it unlikely that recombination caused the significant results. First, the tests using PAML are based on the coding region alone, for which we found no recombination. Second, our estimates of *C* for the entire region were low (*C* = 0.002). Anisimova et al. [67] showed that a LRT comparing M7 to M8 showed no difference in the rate of false positives when *C* = 0.001 than when *C = *0. While our estimate of recombination is higher than this, our p-values (p <0.001) were well below a significance level of 5%. Nevertheless, we cannot entirely rule out the possibility that non-selective forces are responsible for the variation found in *HTR1A*.

### Population substructure and incongruent gene trees

While the results of the evolutionary analyses are interesting and suggestive of the importance of *HTR1A*, several other features of *HTR1A* warrant further discussion, in particular the unusual placement of species and individuals within the haplotype tree. The first is the outlying *M. fascicularis* individual. Despite there being evidence of recombination occurring around the gene, this individual did not share any of the polymorphisms that distinguished the other *M. fascicularis* samples and instead showed greater similarity to the *M. nemestrina*, *M. assamensis*, and *M. silenus* samples. This may be the result of either substructure within *M. fascicularis* or introgression. If the outlier is due to introgression, then this *M. fascicularis* individual is not representative of the entire *M. fascicularis* species, and removal of the outlier from analyses is warranted. However, if the individual outlier is due to population substructure, this suggests that the level of within-species genetic variation for *HTR1A* is extremely high in *M. fascicularis* compared to other species, which could be the result of balancing selection. Two substitutions that separate the outlier from the rest of the *M. fascicularis* individuals are a G/C and a C/A mutation at positions 985 and 986 of the exon, respectively, resulting in the substitution of Ala for His at amino acid 329, which is part of the third intracellular loop of the receptor [56]. Mutations in this part of the receptor are known to affect transductional properties [68], thus the genetic variation present in *M. fascicularis* may be impacting phenotype. What is needed is a broader sampling of *M. fascicularis*, from a variety of geographical regions, to address the possibility of substructure and balancing selection.

The second unusual result indicated by the haplotype tree of *HTR1A* is the placement of the Sulawesi macaques with the *mulatta* group, which includes *M. mulatta*, *M. fuscata*, and *M. cyclopis*, despite the fact that these species are not closely related (see Figure 2). Furthermore, these two groups of species share several derived features not seen in the other macaque species or the vervet, including a complete codon loss and two nonsynonymous mutations, which may have an impact on the receptors’ functionality. When a gene tree is incongruent with a species tree, there are two common explanations. The first is introgression. This seems unlikely based on the geographic distribution of the *mulatta* species and the Sulawesi species. Furthermore, previous studies examining mitochondrial, Y-chromosomal, and autosomal DNA have not shown evidence for introgression between these two groups [24,50]. The second explanation, deep coalescence, is more probable. Deep coalescence occurs when the genetic variation that exists between species was present in the ancestral population. While the processes guiding the subsequent sorting of genetic variants among lineages are often thought of as random, deep coalescence creates a situation that allows for parallel evolution. In particular, if species that are not closely related are subjected to similar selective pressures *and* share genetic variation due to deep coalescence, this would create incongruous trees for the genes under selection.

Because our results suggest that natural selection played a significant role in evolutionary history of *HTR1A*, it is possible that the placement of the Sulawesi species with the *mulatta* species is in fact a product of those selection processes. Unfortunately, until future studies can determine the phenotypic effect of the polymorphisms identified here, the exact cause of the genetic variation of *HTR1A* remains speculative. The grouping of the Sulawesi macaques with the *mulatta* group might suggest that they experienced similar selective pressures with regard to the serotonin system. However, these groups are quite distinct both behaviorally and ecologically [28,69]. Studies on the serotonin system have shown that it is connected with behaviors characteristic of invasive species [5-15,33-35] and it has been argued that genetic variation in serotonin functioning may allow a species to exploit more habitats [31], potentially through increased plasticity [70,71]. Outside of humans, *M. mulatta* has the widest geographic distribution of all primates and their fossil record indicates a long history of expansion [23]. In a similar manner, the geographic location of the Sulawesi macaques are of interest as they have crossed the Makassar Straits between Borneo and Sulawesi and thus Wallace's line at least once [72], something that very few mammalian species have managed to do. Nevertheless, this explanation is also problematic as both *M. nemestrina* and *M. fascicularis* are also adept dispersers [73,74] and yet do not group with the Sulawesi macaques or the *mulatta* group, and as of yet there is no direct evidence to support this hypothesis. Thus, although our results indicate an unusual evolutionary history for *HTR1A*, at this stage it is unclear as to its exact cause.

## Conclusions

The pattern of variation found in the two genes examined, *HTR1A* and *HTR1B*, show that each gene was influenced by different evolutionary forces and that, for *HTR1A*, positive selection seemed to have played an important role. In particular, two independent lineages of macaques, the *mulatta* group and the Sulawesi group, show remarkable similarity to each other for *HTR1A*, to the exclusion of more closely related species, which may be the result of having experienced similar selective pressures. Overall, selection on *HTR1A* has led to a high level of divergence among species, and this may explain some of the species differences in behavior, including aggression, age at dispersal, and exploratory behavior, although further studies on the phenotypic effect of the sequenced polymorphisms are needed to confirm this. It has been hypothesized that parallel evolution has acted on the serotonin system of both macaques and humans to allow both taxa to successfully exploit a diverse set of habitats over large regions of the world. Because previous research has shown that selection has acted on the serotonin system in humans as well, this study provides support for this hypothesis and opens the door for future studies examining the interaction between genetics, serotonin, and behavior.

## Methods

### Subjects

We used aliquots of previously extracted DNA from 20 *Macaca mulatta* (11 from India, 9 from China), 11 *M. fascicularis*, 11 *M. fuscata*, 6 *M. nemestrina*, and 4 *M. sylvanus* individuals (Additional file 1: Table S1). Previous studies [24,49-53] have shown that *M. mulatta* consists of two groups that are genetically distinct, roughly split between those of Indian and Chinese origin. Because the substructure of this species could influence our analyses, we analyzed each population separately as well as the species as a whole. In addition to the previously mentioned samples, one sample each from *Macaca assamensis*, *M. cyclopis*, *M. nigra*, *M. silenus* and *Chlorocebus aethiops* (Additional file 1: Table S1) were used in PAML analyses (see below) to help place the results in a phylogenetic framework. Because of unusual results obtained for *M. nigra* for *HTR1A*, for post hoc analyses on *HTR1A* we obtained 2 additional *M. nigra* samples, 1 *M. maura*, and 1 *M. tonkeana*. In total, 61 individuals were used producing 122 (=2N) haplotypes. The 11 different macaque species analyzed span the range of behavioral variation found in the genus. The *C. aethiops* (vervet) sample was used as an outgroup for the macaque species.

The individuals used in this study have been used in previous research and more detailed information can be found in [51,53,75]. All primates providing samples for this study were managed in accordance with the United States Public Health Service Policy on the Humane Care and Use of Laboratory Animals. Efforts were made to select nonrelatives that originated from a variety of geographic regions; these decisions were based on breeding records or single-nucleotide polymorphism (SNP)/short tandem repeat analyses [53]. Samples that indicated possible hybridization were avoided, including between Chinese and Indian populations of *M. mulatta*.

### PCR and sequencing

In total, we sequenced 3,119 bp for *HTR1A* and 2,117 bp for *HTR1B*. For both genes, we amplified the entire coding region as well as the flanking regions, including potential regulatory regions, using both previously published [76] and newly designed primers (Additional file 1: Table S2). The new primers were designed based on the *Macaca mulatta* draft assembly [40] using Primer3 [77] and GeneRunner [78]. In addition to *HTR1A* and *HTR1B*, five noncoding regions from across the macaque genome were amplified in all of the samples (Additional file 1: Table S3) and used for the HKA test (see below) [79]. These regions have no known function and are thus referred to in this paper as the “nonfunctional regions,” so as to differentiate these regions from the noncoding regions surrounding the serotonin genes, the latter of which may have potential regulatory roles. Because these nonfunctional regions are at least 20,000 base pairs from the nearest coding region [80], they are presumed to be evolving neutrally. The PCR protocols differed for each of the regions amplified and are available upon request. The sequence data for *HTR1A* and *HTR1B* are available online [GenBank: KC862384-KC862501]. For the five nonfunctional regions, see [53].

We used ExoSAP-IT to clean up the PCR product and submitted it to the W.M. Keck Center for Comparative and Functional Genomics, UIUC for Sanger sequencing. As with the PCR primers, the sequencing primers used were a combination of published and newly designed primers. Efforts were made to design primers that would provide substantial overlap with each other so that any one region being analyzed would have multiple reads from different primers, ensuring the accuracy and quality of the sequence.

Sequences were aligned and edited manually using Sequencher [81]. Each heterozygote base pair was confirmed visually by identifying clear double peaks in the chromatogram. All SNPs (single nucleotide polymorphisms) and indels (insertions/deletions) were identified. Where indels involved more than one sequential nucleotide, the entire deleted region was treated as a single mutational event. Haplotypes were determined using the program Phase v1 [82-84]. In order to visualize the genetic variation and relationships among the haplotypes of each gene, we constructed a haplotype tree using the reduced median method in Network v4.5 (fluxus-engineering.com). All samples sequenced were used to create the tree. Two haplotype trees were created for each gene: one showing the entire region sequenced (both coding and noncoding) and one showing the coding region only.

### Analysis

With the exception of the likelihood ratio tests and the haplotype networks, all analyses were conducted on the five species for which we had multiple samples: *M. mulatta*, *M. fuscata*, *M. fascicularis*, *M. nemestrina*, and *M. sylvanus*. Several indices of molecular diversity were calculated for each of the five species using Arlequin [85]. These included two different estimates of theta (θ = 4Nμ, where N is the effective population size and μ is the mutation rate): θ_S_ [86] and θ_π_ [87], which were used to estimate within-species diversity. Nucleotide diversity (averaged over all loci) [87] was used to estimate within- and between- species diversity.

In order to determine whether selection has acted on either gene, we used multiple approaches. For all selection tests, we used a significance level of 0.05. First, we applied the HKA test, which compares the ratio of within- versus between-species variation in a gene of interest with that of several unlinked, neutral loci. A gene that is evolving neutrally should not have a ratio that varies significantly from that of the other loci considered. We used the five nonfunctional regions described above and in Table S3 (Additional file 1) for comparison to the serotonin genes in the HKA test. In order to get an idea of where selection is occurring, in addition to examining the entire region sequenced for each gene, we also carried out the HKA tests on the coding region alone. Because the HKA test requires a comparison between two species, we conducted pairwise comparisons for all five species using software provided by J. Hey [88]. We also compared each of the five species to the vervet, which is an outgroup to macaques. For each test we ran 10,000 simulations based on parameters estimated from the data to obtain a simulated distribution of the X^2^ test statistic. P-values were obtained by comparing the observed X^2^ statistic with the simulated distribution.

Second, we examined the ratio of nonsynonymous to synonymous mutations (**ω**) in the coding regions of the genes. Positive selection increases the relative rate of nonsynonymous substitutions, whereas purifying selection decreases it [89]. For this approach, we used multiple tests. We used the program Mega v.4 [90] to determine whether **ω** between any two species significantly differs from 1.0 [89,91]. If **ω** >1.0, this indicates positive selection, while **ω** <1.0 indicates purifying selection. However, this on its own is a very conservative test as there is expected to be a limit to the number and locations of mutations that a gene can maintain while still remaining functional. This means that **ω** will often be low (**ω** <1.0) even in the presence of positive selection. Therefore, we conducted likelihood ratio tests (LRT) on all of the samples available using the program PAML (Phylogenetic Analysis using Maximum Likelihood) [92,93]. PAML determines the maximum likelihood values for different models of evolution within a phylogenetic framework. Models that incorporate positive selection can be compared to null models and the LRT used to determine which model best fits the data. Because PAML requires only one sequence per species, we included all ten species in the LRT using the phylogeny seen in Figure 2 [62]. For this study, we examined two categories of evolutionary models. First, we tested various branch models, which allow **ω** to vary among each of the branches of the macaque phylogeny, to see if selection has occurred on a specific lineage [94]. Tested in this way, **ω** does not have to be greater than one, but simply elevated compared to other lineages of the macaque phylogeny. Second, we compared several sites models using PAML: M1a vs. M2a, M7 vs. M8, and M8a vs. M8 (see [95-97] for detailed explanation of models). These models allow **ω** to vary among different sites on the gene using different parameters [95,96,98]. Most sites on a gene are under strong purifying selection, which reduces the overall **ω** for a gene and can hide signals of positive selection occurring only in a small portion of the gene. Testing the sites models in PAML can determine if a portion of a gene shows signs of positive selection, even if the average **ω** ratio over the entire gene is low. Specifically, models M1a, M7, and M8a are null models which assume that there are no class of sites within the gene where **ω** >1.0 (no positive selection). Models M2a and M8 are similar to the null models except that they allow for an additional category of sites where **ω** >1.0 (positive selection). For these selection models, a Bayes empirical Bayes test was used to determine the posterior probability that a given site fell into the category where **ω** >1.0 [97]. A posterior probability of 95% was considered significant.

Finally, using Arlequin, we calculated Tajima’s D for all five species, which detects skews in the frequency spectrum of alleles by comparing two different estimates of theta, θ_S_ and θ_π_ [99]. An excess of rare alleles (θ_S_ > θ_π_, leading to significantly negative Tajima's D) is consistent with positive selection, while an excess of intermediate-frequency alleles (θ_S_ < θ_π_, leading to significantly positive Tajima's D) is consistent with balancing selection.

Many of the tests that we employed assume absence of recombination, which can affect the results. Specifically, violation of this assumption can increase false positives in the LRT of the sites models using PAML [67]. In contrast, the assumption of no recombination is conservative for the HKA test [79]. We used the SITES program of J. Hey [88] to obtain an estimate of the recombination parameter C, where C = 2Nc, and c is the rate of recombination per generation per base pair and N is the effective population size [60]. Because the LRT only examines the coding region, whereas the HKA test can analyze both coding and noncoding regions, we obtained estimates of C based on the entire regions sequenced, and on the coding region alone. Presence of recombination indicates that a significant result for the LRT of the sites models should be treated with caution, but should not affect the interpretation of a significant result for HKA.

## Additional file

Additional file 1: Figure S1. Haplotype networks for the coding regions only of *HTR1A* (top) and *HTR1B* (bottom). **Table S1.** List of sources for species samples. **Table S2.** List of primers used to sequence *HTR1A* and *HTR1B*. **Table S3.** Additional noncoding loci sequenced. **Table S4.** Genetic distance within and between species for the five nonfunctional regions sequenced. **Table S5.** Indices of within-species genetic diversity found in the five nonfunctional regions sequenced. **Table S6.** Results of the HKA test using only the coding region for *HTR1A*. **Table S7.** Results of the HKA test when using *Chlorocebus aethiops* as the comparative group. **Further Discussion**.
