# Supplementary material for: Patterns of genetic variation and the role of selection in HTR1A and HTR1B in macaques (Macaca)
Source: BMC Genet. 2014 Nov 7;15:116. doi: 10.1186/s12863-014-0116-5 (PMC4228068; doi:10.1186/s12863-014-0116-5)
Supplement: Additional file 1: Figure S1. — Haplotype networks for the coding regions only of HTR1A (top) and HTR1B (bottom). Table S1. List of sources for species samples. Table S2. List of primers used to sequence HTR1A and HTR1B. Table S3. Additional noncoding loci sequenced. Table S4. Genetic distance within and between species for the five nonfunctional regions sequenced. Table S5. Indices of within-species genetic diversity found in the five nonfunctional regions sequenced. Table S6. Results of the HKA test using only the coding region for HTR1A. Table S7. Results of the HKA test when using Chlorocebus aethiops as the comparative group. Further Discussion. [file 12863_2014_116_MOESM1_ESM.doc]

**Additional File**

**
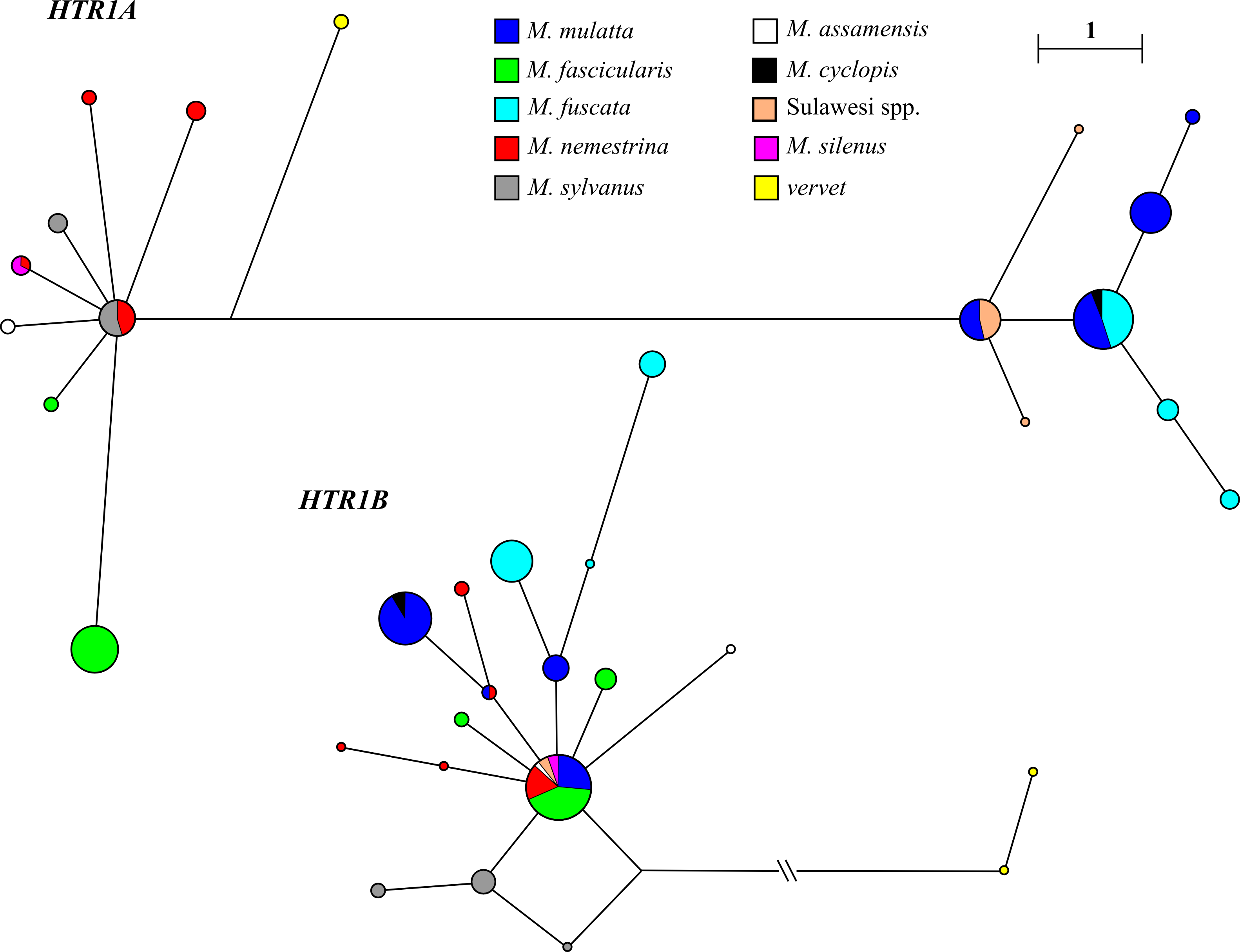
**

**Figure S1.** Haplotype networks for the coding regions only of *HTR1A* (top) and *HTR1B* (bottom). Each circle represents a haplotype whose size is proportional to the frequency of the haplotype. The lengths of the lines connecting the circles are proportional to the number of mutations that separate each haplotype, according to the scale provided. Because of the large separation of the vervet from the macaques in *HTR1B* (35 mutations), this line is not drawn to scale. Sulawesi species include *M. nigra*, *M. tonkeana*, and *M. Maura*. For *HTR1B*, only the original *M. nigra* is shown (see **METHODS** and **RESULTS**).

**Table S1.** List of sources for species samples. Number of samples indicates the number of individuals for which we were able to obtain complete sequence data for both genes. *See [1].

| **Species** | | **N** | **Source** |
| --- | --- | --- | --- |
| *M. mulatta* | | 20 | DG Smith and J Satkoski, UC Davis |
|  | Indian | 11 | DG Smith and J Satkoski, UC Davis |
|  | Chinese | 9 | DG Smith and J Satkoski, UC Davis |
| *M. fascicularis* | | 11 | DG Smith and J Satkoski, UC Davis |
| *M. fuscata* | | 11 | Oregon National Primate Research Center*; A Deinard |
| *M. nemestrina* | | 6 | DG Smith, UC Davis; New Iberia National Primate Research Center |
| *M. sylvanus* | | 4 | DG Smith, UC Davis; A Deinard |
| *M. assamensis* | | 1 | New Iberia National Primate Research Center |
| *M. cyclopis* | | 1 | New England National Primate Research Center |
| *M. nigra* | | 3 | Baltimore Zoo; D. Melnick |
| *M. silenus* | | 1 | Woodland Park Zoo |
| *M. maura* | | 1 | D Melnick |
| *M. tonkeana* | | 1 | D Melnick |
| *C. aethiops* | | 1 | New Iberia National Primate Research Center |
| *Total* | | 61 |  |

**Table S2.** List of primers used to sequence *HTR1A* and *HTR1B*. For both genes, the number in the primer name represents the position of the primer relative to the start of the coding region, with +1 marking the first nucleotide of the start codon. For *HTR1A*, these numbers are based on the rhesus macaque reference genome. For *HTR1B*, we followed the numbering system of Cigler et al. [2], which is based on the human genome. For the source column: a = primers we designed for this project; b = primers published Cigler et al. [2].

| **Gene** | **Primer ID** | **Sequence** | **Type** | **Source** |
| --- | --- | --- | --- | --- |
| *HTR1A* | HTR1A_-433F | 5'-ACA GAG TGA CCG TGG AGG ATG-3' | PCR and Sequencing | a |
|  | HTR1A_-309F | 5'-AGC GAC AGA CAG ACG TTC C-3' | Sequencing | a |
|  | HTR1A_18F | 5'-TGG TCA GGG CAA CAA CAC-3' | Sequencing | a |
|  | HTR1A_272R | 5'-ATG GGC AGC ACT AAC ACC-3' | Sequencing | a |
|  | HTR1A_-107R | 5'-TCG GAG GAA GGG AAT GCA G-3' | Sequencing | a |
|  | HTR1A_621R | 5'-CAG CGG GAT GTA GAA AGC-3' | Sequencing | a |
|  | HTR1A_328F | 5'-CTG TTT ATC GCC CTG GAC-3' | Sequencing | a |
|  | HTR1A_479F | 5'-GGC TTA TTG GCT TCC TCA TC-3' | Sequencing | a |
|  | HTR1A_1074R | 5'-AAG CCA GCA GAG GAT GAA GG-3' | Sequencing | a |
|  | HTR1A_1262R | 5'-TGG CGG CAG AAC TTA CAC-3' | Sequencing | a |
|  | HTR1A_942F | 5'-TTG TGC CTC CGC CTC TTT-3' | Sequencing | a |
|  | HTR1A_1523R | 5'-GCA AAG TCT GAG CCA ATG TC-3' | Sequencing | a |
|  | HTR1A_1187F | 5'-CGG TCA TTT ACG CAT ACT TC-3' | Sequencing | a |
|  | HTR1A_1364F | 5'-CTT CTT CTC TGT CTC TCT GCT C-3' | Sequencing | a |
|  | HTR1A_1954F | 5'-CCC TGC TTC CTT TGT TTC-3' | Sequencing | a |
|  | HTR1A_2212R | 5'-GCC TCC CGC AGT AAG TAA GTG-3' | PCR and Sequencing | a |
|  | HTR1A_-1071F | 5'-AGT GCA GTG GCG CGA GAA-3' | PCR and Sequencing | a |
|  | HTR1A_-1059F | 5'-CGA GAA CGG AGG GAG GTA AC-3' | Sequencing | a |
|  | HTR1A_-660R | 5'-AGT GCC TCT TTC CTC TGG-3' | Sequencing | a |
|  | HTR1A_-744R | 5'-TCA GAA CTC ACT TAC ACA CAC C-3' | Sequencing | a |
|  | HTR1A_-530R | 5'-CAC TTG CCT TCC CTT TCA GT-3' | PCR and Sequencing | a |
| *HTR1B* | HTR1B_1247R | 5'-TTC GAC CTA CCT GTG GAA CC-3' | PCR and Sequencing | b |
|  | HTR1B_-174F | 5'-GGC TGC CGC ACC CAT GAC CT-3' | Sequencing | b |
|  | HTR1B_864F | 5'-CCA AGT CAA AGT GCG AGT CT-3' | Sequencing | b |
|  | HTR1B_-40R | 5'-ATG GAG CGG ACG AAG GAG A-3' | Sequencing | b |
|  | HTR1B_493R | 5'-TCT TGG GAG TCC TTT TAG C-3' | Sequencing | b |
|  | HTR1B_317F | 5'-GCA CCA TGT ACA CTG TCA CC-3' | Sequencing | a |
|  | HTR1B_-595F | 5'-CAG CGC TGC TCC TAG ACT TC-3' | PCR and Sequencing | b |
|  | HTR1B_957F | 5'-TTT GGG AGC CTT TAT TGT G-3' | PCR and Sequencing | a |
|  | HTR1B_1385R | 5'-TGG GCA GGG AAG TTC TAC-3' | Sequencing | a |
|  | HTR1B_1231F | 5'-TCC ACA GGT AGG TCG AAT C-3' | Sequencing | a |
|  | HTR1B_1536R | 5'-TGG TTC TAG TGG GCA TTA TC-3' | PCR and Sequencing | a |

**Table S3.** Additional noncoding loci sequenced. The nearest feature was identified by blasting the consensus sequence against Build 1.1 (annotated) of the rhesus genome on the NCBI website.

| **Chromosome** | **Name** | **Length** | **Nearest Feature (base pairs)** |
| --- | --- | --- | --- |
| 4 | Chr04-2 | 478 bp | 69,159 at 5' side: similar to elongation factor 1 epsilon-1 |
| 6 | Chr06-5 | 428 bp | 77,112 at 5' side: hypothetical protein |
| 9 | Chr09-2 | 526 bp | None within 1Mb |
| 14 | Chr14-2 | 462 bp | 68,130 at 5' side: CD82 molecule isoform 1 |
| 20 | Chr20-1 | 400 bp | 20,964 at 3' side: similar to xylosyltransferase I |

**Table S4.** Genetic distance within and between species for the five nonfunctional regions sequenced. The diagonal elements show the nucleotide diversity within species and the off-diagonal elements show the nucleotide diversity between species. For ease of comparative purposes, we highlighted all areas where the nucleotide diversity of *HTR1A* was relatively lower than in the nonfunctional regions. For the ratio of nucleotide diversity of nonfunctional regions to *HTR1A*, x, light gray = 1.05 < x ≤ 1.5; medium gray = 1.5 < x ≤ 3; dark grey = 3 < x. Ratios were determined using the nucleotide diversity calculated when outlier was removed. Mul = *M. mulatta*; Fas = *M. fascicularis*; Fus = *M. fuscata*; Nem = *M. nemestrina*; Syl = *M. sylvanus*.

|  |  | Mul | Fas | Fus | Nem | Syl |
| --- | --- | --- | --- | --- | --- | --- |
| Chr04-2 | Mul | 0.0031 |  |  |  |  |
|  | Fas | 0.0037 | 0.0036 |  |  |  |
|  | Fus | 0.0051 | 0.0059 | 0.0020 |  |  |
|  | Nem | 0.0019 | 0.0021 | 0.0038 | 0.0000 |  |
|  | Syl | 0.0122 | 0.0119 | 0.0142 | 0.0104 | 0.0000 |
| Chr06-5 | Mul | 0.0034 |  |  |  |  |
|  | Fas | 0.0043 | 0.0039 |  |  |  |
|  | Fus | 0.0023 | 0.0032 | 0.0009 |  |  |
|  | Nem | 0.0038 | 0.0045 | 0.0024 | 0.0037 |  |
|  | Syl | 0.0019 | 0.0026 | 0.0006 | 0.0019 | 0.0000 |
| Chr09-2 | Mul | 0.0065 |  |  |  |  |
|  | Fas | 0.0080 | 0.0050 |  |  |  |
|  | Fus | 0.0077 | 0.0083 | 0.0007 |  |  |
|  | Nem | 0.0070 | 0.0049 | 0.0068 | 0.0034 |  |
|  | Syl | 0.0279 | 0.0251 | 0.0305 | 0.0267 | 0.0000 |
| Chr14-2 | Mul | 0.0042 |  |  |  |  |
|  | Fas | 0.0062 | 0.0049 |  |  |  |
|  | Fus | 0.0043 | 0.0038 | 0.0006 |  |  |
|  | Nem | 0.0029 | 0.0044 | 0.0027 | 0.0004 |  |
|  | Syl | 0.0063 | 0.0078 | 0.0068 | 0.0045 | 0.0000 |
| Chr20-1 | Mul | 0.0024 |  |  |  |  |
|  | Fas | 0.0037 | 0.0043 |  |  |  |
|  | Fus | 0.0017 | 0.0029 | 0.0009 |  |  |
|  | Nem | 0.0023 | 0.0036 | 0.0015 | 0.0018 |  |
|  | Syl | 0.0183 | 0.0198 | 0.0180 | 0.0185 | 0.0000 |

**Table S5.** Indices of within-species genetic diversity found in the five nonfunctional regions sequenced. See Table S4 for species names. For *M.* *mulatta*, indices for both the Chinese and the Indian populations are shown separately, as well as indices for the species as a whole. Because there were no polymorphisms for *M. sylvanus*, it is not listed in this table.

|  |  |  | **Mul** | | | **Fas** | **Fus** | **Nem** |
| --- | --- | --- | --- | --- | --- | --- | --- | --- |
|  |  |  | China | India | Total |  |  |  |
| **Chr04-2** | Polymorphisms | | 6 | 5 | 6 | 10 | 3 | 0 |
|  | Theta (S) | | 1.61 | 1.51 | 1.41 | 2.35 | 0.85 | --- |
|  | Theta (π) | | 1.50 | 1.51 | 1.51 | 1.72 | 0.94 | --- |
|  | Tajima's D | | -0.21 | 0.00 | 0.19 | -0.80 | 0.30 | --- |
|  |  | p-value | > 0.1 | > 0.1 | > 0.1 | > 0.1 | > 0.1 | --- |
| **Chr06-5** | Polymorphisms | | 9 | 4 | 9 | 7 | 1 | 0 |
|  | Theta (S) | | 2.41 | 1.21 | 2.12 | 1.65 | 0.28 | --- |
|  | Theta (π) | | 1.51 | 1.13 | 1.45 | 1.68 | 0.39 | --- |
|  | Tajima's D | | -1.23 | -0.21 | -0.92 | 0.05 | 0.72 | --- |
|  |  | p-value | > 0.1 | > 0.1 | > 0.1 | > 0.1 | > 0.1 | --- |
| **Chr09-2** | Polymorphisms | | 7 | 17 | 19 | 14 | 2 | 4 |
|  | Theta (S) | | 1.87 | 5.12 | 4.47 | 3.42 | 0.56 | 1.41 |
|  | Theta (π) | | 1.81 | 5.25 | 3.46 | 2.68 | 0.37 | 1.83 |
|  | Tajima's D | | -0.10 | 0.10 | -0.74 | -0.71 | -0.81 | 1.11 |
|  |  | p-value | > 0.1 | > 0.1 | > 0.1 | > 0.1 | > 0.1 | > 0.1 |
| **Chr14-2** | Polymorphisms | | 7 | 8 | 11 | 9 | 1 | 1 |
|  | Theta (S) | | 1.87 | 2.52 | 2.62 | 2.14 | 0.31 | 0.35 |
|  | Theta (π) | | 1.67 | 2.08 | 1.94 | 2.27 | 0.26 | 0.20 |
|  | Tajima's D | | -0.34 | -0.66 | -0.80 | 0.18 | -0.34 | -1.11 |
|  |  | p-value | > 0.1 | > 0.1 | > 0.1 | > 0.1 | > 0.1 | > 0.1 |
| **Chr20-1** | Polymorphisms | | 5 | 2 | 6 | 8 | 2 | 2 |
|  | Theta (S) | | 1.34 | 0.60 | 1.41 | 1.88 | 0.55 | 0.71 |
|  | Theta (π) | | 1.19 | 0.45 | 0.95 | 1.71 | 0.35 | 0.71 |
|  | Tajima's D | | -0.32 | -0.65 | -0.87 | -0.26 | -0.84 | 0.02 |
|  |  | p-value | > 0.1 | > 0.1 | > 0.1 | > 0.1 | > 0.1 | > 0.1 |

**Table S6.** Results of the HKA test using only the coding region for *HTR1A*. Lower left diagonal shows the sum of deviations calculated in the HKA program and the upper right shows their respective p-values. Numbers bolded have a p < 0.05.

| ***HTR1A*: all samples** | | | | | |
| --- | --- | --- | --- | --- | --- |
|  |  |  |  |  |  |
|  | Mul | Fas | Fus | Nem | Syl |
| Mul | --- | 0.099 | 0.876 | **0.051** | **0.07** |
| Fas | 11.55 | --- | 0.35 | 0.115 | 0.171 |
| Fus | 4.19 | 9.28 | --- | 0.172 | 0.405 |
| Nem | **13.47** | 9.15 | 11.48 | --- | 0.258 |
| Syl | 14.24 | 11.21 | 7.46 | 8.38 | --- |
|  |  |  |  |  |  |
| ***HTR1A*: outlier removed** | | | | | |
|
|  | Mul | Fas | Fus | Nem | Syl |
| Mul | --- | **0.008** | 0.876 | **0.051** | **0.07** |
| Fas | **18.80** | --- | **0.037** | **0.016** | **0.019** |
| Fus | 4.19 | **16.29** | --- | 0.172 | 0.405 |
| Nem | **13.47** | **14.22** | 11.48 | --- | 0.258 |
| Syl | **14.24** | **20.19** | 7.46 | 8.38 | --- |
|  |  |  |  |  |  |
| ***HTR1B*** | | | | | |
|
|  | Mul | Fas | Fus | Nem | Syl |
| Mul | --- | 0.955 | 0.411 | 0.167 | **0.003** |
| Fas | 1.91 | --- | 0.693 | **0.024** | **0.004** |
| Fus | 8.37 | 6.07 | --- | 0.455 | 0.101 |
| Nem | 8.75 | **9.31** | 7.78 | --- | 0.145 |
| Syl | **27.14** | **28.48** | 13.42 | 11.29 | --- |

**Table S7.** Results of the HKA test when using *Chlorocebus aethiops* as the comparative group.

|  |  | ***HTR1A*** | |  | ***HTR1B*** | |
| --- | --- | --- | --- | --- | --- | --- |
|  |  | Sum of dev. | p-value |  | Sum of dev. | p-value |
| *M. mulatta* | | 5.78 | 0.544 |  | 13.70 | 0.060 |
| *M. fascicularis* | | 5.85 | 0.551 |  | 13.86 | 0.054 |
|  | outlier removed | 8.96 | 0.224 |  | --- | --- |
| *M. fuscata* | | 6.57 | 0.438 |  | 9.22 | 0.236 |
| *M. nemestrina* | | **16.60** | **0.030** |  | **18.64** | **0.022** |
| *M. sylvanus* | | 7.39 | 0.335 |  | 7.85 | 0.338 |

**FURTHER DISCUSSION**

**Comparison of nonfunctional regions to *HTR1A***

Table S4 provides the genetic diversity found within and between species in the five nonfunctional regions sequenced and used in the HKA test. The highlighted cells indicate where genetic diversity was higher than that found in *HTR1A*. An examination of this table shows that the highlighted cells commonly occur along the diagonal elements. That is, *HTR1A* shows relatively *low* *intraspecific* variation compared to other areas of the genome. In contrast, for the off-diagonal elements, *HTR1A* is always higher (showing relatively *high* *interspecific* variation) except for most comparisons with *M. sylvanus*, or between *M. mulatta* and *M. fuscata*. This is entirely consistent with the results of the HKA test.

**Additional HKA results**

When looking at the coding region alone, the results of the HKA test largely replicated what was seen when examining the entire regions sequenced. There were a few exceptions to this. First for *HTR1A*, comparisons of the *mulatta* group to *M. sylvanus* became significant, providing further support to the idea that selection is occurring in *HTR1A*. For *HTR1B*, three comparisons became significant: that between *M. mulatta* and *M. sylvanus*, that between *M. fascicularis* and *M. sylvanus*, and that between *M. fascicularis* and *M. nemestrina*. The significant result for *M. nemestrina* and *M. fascicularis* is unusual since the sum of deviations was not particularly high (9.31). We based our p-value (p = 0.024) on 10,000 simulations based on parameters estimated from the data to obtain a simulated distribution of the X^2^ test statistic. However, based on a traditional chi-square distribution, the p-value would be p = 0.503. While there were always differences between p-values based on the simulated distribution and those based on the chi-squared distribution, they were usually small. This is the only instance where the two values displayed such large differences, and thus it is difficult to interpret this result.

In regards to the significant results for comparisons of *M. sylvanus* to both *M.* *mulatta* and *M. fascicularis*, these results are interesting because, unlike the HKA results of *HTR1A*, they were driven by a higher than expected level of polymorphisms within *M. sylvanus*. This might be interpreted as evidence for balancing selection with *M. sylvanus* for the coding region. However, this is based on just two polymorphisms, both of which are synonymous. Furthermore, as this is the only evidence provided for selection happening within macaques species for *HTR1B*. So despite the strong p-values, it is not very clear that this is a true signal of selection.

When applying the HKA to the serotonin receptor genes using the vervet (*C. aethiops*) as the comparative group, we got different results then when comparisons were made within the macaque species. Overall, evidence of selection is much weaker. For *HTR1B*, one comparison (that between the vervet and *M. nemestrina*) was significant (p = 0.022), and two other comparisons showed only borderline significance (*M. fascicularis*: p = 0.054; *M. mulatta*: p = 0.06) (Table S7). In contrast, only *M. nemestrina* showed a significant result (p = 0.030) when looking at *HTR1A*. One might interpret this as mild evidence for a selective sweep that occurred on *HTR1B* in the macaque genus, perhaps after *M. sylvanus* had emerged, but before the other species had fully speciated. However, even if the results were stronger, this would not help to explain the large amount of behavioral differences that occur between macaque species. Nor is it clear, with just a singular vervet sample, whether it was the macaques that were the target of selection, versus the vervet. Additional sampling of vervet individuals and other Old World monkeys would be need to address that question.

REFERENCES

1. Ferguson B, Capitanio J, Folks T, Hotchkiss C, Johnson Z, Kean L, Kubisch HM, Lank S, Lyons L, Miller GM, Nylander J, O'Connor D, Vallender EJ, Wiseman R: **Resource brief: the National Non-Human Primate DNA Bank.** *Methods* 2009, **49**(1)**:**3-4.

2. Cigler T, LaForge KS, McHugh PF, Kapadia SU, Leal SM, Kreek MJ: **Novel and previously reported single-nucleotide polymorphisms in the human 5-HT1B receptor gene: No association with cocaine or alcohol abuse or dependence.** *Am J Med Genet* 2001, **105**(6)**:**489-497.
